# Successful radical resection of pancreatic head carcinoma in a patient with replaced right hepatic artery originating from posterior inferior pancreaticoduodenal artery: a case report

**DOI:** 10.1186/s40792-017-0352-9

**Published:** 2017-06-19

**Authors:** Yusuke Sakamoto, Takahisa Fujikawa, Akira Tanaka

**Affiliations:** Department of Surgery, Kokura Memorial Hospital, 3-2-1 Asano, Kokurakita-ku, Kitakyushu, Fukuoka 802-8555 Japan

**Keywords:** Pancreatic head cancer, Replaced right hepatic artery, Pancreaticoduodenectomy, Preoperative anatomical assessment

## Abstract

Anatomical variations of hepatic arteries may be problematic in pancreaticoduodenectomy (PD). We experienced pancreatic head cancer in a patient with rare variation of hepatic artery and performed PD successfully with the resection of this artery. A 75-year-old woman showed pancreatic head tumor on CT. Preoperative CT detected rare variation of hepatic artery; posterior segmental branch of right hepatic artery (RHA-PB) originating from posterior inferior pancreaticoduodenal artery. The image also demonstrated that there was a junction between RHA-PB and anterior branch of right hepatic artery (RHA-AB). We performed PD for suspected pancreatic head cancer. We divided RHA-PB for complete resection of cancer because we preoperatively knew that there was the junction between RHA-PB and RHA-AB. She was discharged uneventfully, and there was no evidence of local recurrence throughout the whole course. Careful preoperative assessment of hepatic blood supply is the key to perform successful PD even in this troublesome situation.

## Background

Anatomical variations of hepatic arteries are of great importance for abdominal surgery and interventional radiology. Especially, aberrant right hepatic artery (RHA) may be problematic in pancreaticoduodenectomy (PD) [1]. Resection of this aberrant artery is sometimes necessary to achieve R0 resection of cancer, although it can lead to possible bile duct and liver ischemia.

We experienced locally advanced pancreatic head cancer in a patient with an unusual aberrant RHA and performed PD successfully by sacrificing this vessel without any complications.

## Case presentation

A 75-year-old woman with a history of surgical intervention for aortic aneurysm showed dilatation of her main pancreatic duct and pancreatic head tumor on CT scan at an annual medical check-up. Endoscopic ultrasound-guided fine needle aspiration of the pancreatic head tumor was performed, and the result was group 4. She was referred to our department for the surgical treatment of suspected pancreatic head cancer.

**Fig. 1:**
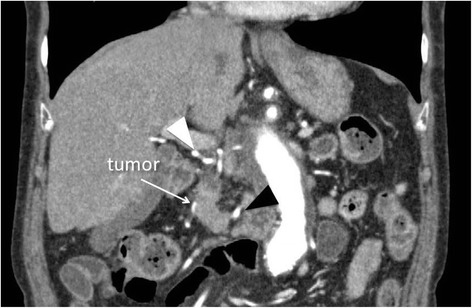
An enhanced CT scan demonstrated there was a tumor (labeled as “tumor”) in the pancreatic head and RHA-PB (*white arrow head*) originating from PIPDA (*black arrow head*) was running through the tumor

**Fig. 2:**
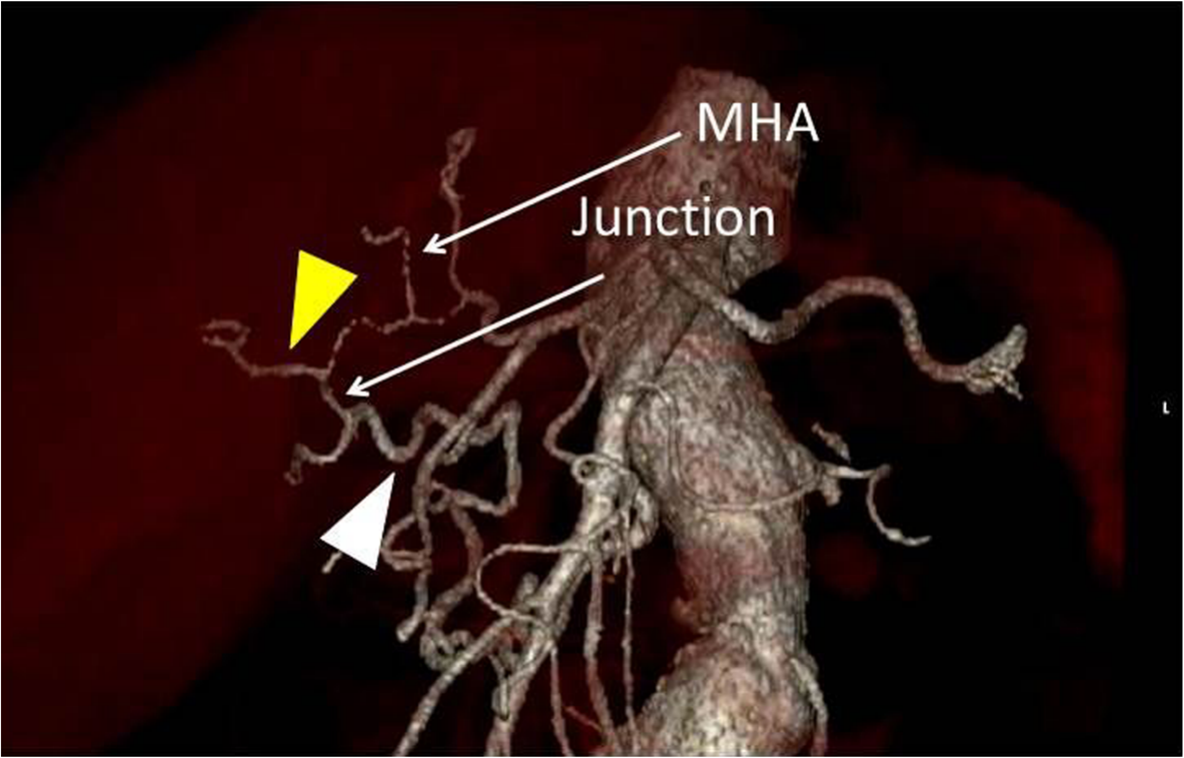
A 3D reconstruction of CT showed there were middle hepatic artery (labeled as “*MHA*”) and a junction (labeled as “junction”) between RHA-PB (*white arrow head*) and RHA-AB (*yellow arrow head*) at the hilar portion

Preoperative enhanced high-resolution CT detected pancreatic head tumor and major posterior segmental branch of RHA (RHA-PB) which originated from posterior inferior pancreaticoduodenal artery (PIPDA) and run through the tumor (Fig. 1). The image also demonstrated that there was a junction between RHA-PB and minor anterior segmental branch of RHA (RHA-AB) at the hilar portion (Fig. 2).

She received PD combined with regional lymph node dissection. At laparotomy, RHA-PB was isolated from the hepatoduodenal ligament and confirmed that it originated from PIPDA in accordance with the preoperative findings, which revealed to run through the tumor. To preserve RHA-PB, we had to preserve posterior pancreaticoduodenal arcade and the common trunk of PIPDA, which decreased the curability of the tumor. We ensured that the color of the liver did not change after clamping of RHA-PB. Finally, we ligated and divided RHA-PB for complete resection of the pancreatic head tumor because we knew from the preoperative images that there was the junction between RHA-PB and RHA-AB at the hilar portion. We performed the operation without any complication and achieved R0 resection macroscopically (duration of surgery, 355 min; blood loss, 220 mL).

**Fig. 3:**
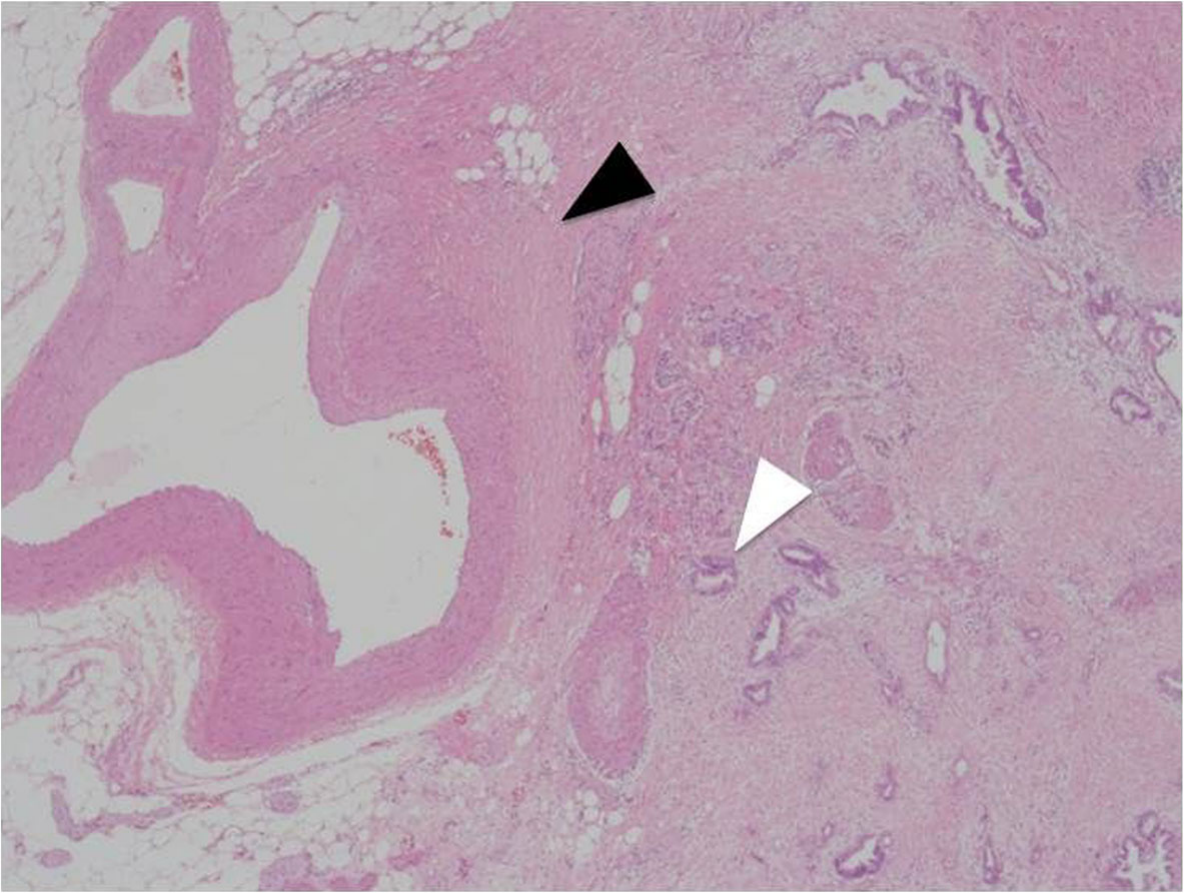
The pathological examination revealed that the tumor (*white arrow head*) invasion was seen near the intraparenchymal vessels (*black arrow head*)

Her postoperative course was uneventful, and she was discharged on POD15 without hepatic or biliary ischemia. The pathological examination revealed that the tumor was diagnosed as an adenocarcinoma of the pancreas. The tumor invasion was seen near the intraparenchymal vessels (Fig. 3), but R0 resection was performed.

Although she died from chronic heart failure 13 months after the operation, local recurrence had not be seen throughout the whole course.

## Discussion

We experienced pancreatic head cancer in a patient with rare variation of hepatic artery; RHA-PB originating from PIPDA. We preoperatively identified this unusual aberrant artery and the junction at the hilar portion, which led to successful PD with the resection of this unusual aberrant artery.

We could not find a report on RHA-PB originating from PIPDA. In 1966, Michels et al. described 10 types of anatomic variations of hepatic artery [2] and many authors commonly used Michels’ classification in examining anatomic variations of hepatic artery. However, several authors reported rare variations which could not be classified according to Michels (Table 1) [3–8]. To the best of our knowledge, rare variations which were not described in Michels’ classification existed in 1.8–18% of cases, and the variation we encountered in this case has not been reported before.

**Table 1:** Hepatic arterial variations incompatible with the descriptions in the Michels’ classification

Author	Number of patients	Type
Ugurel MS	1	RHA originating from the middle colic artery
	1	RHA originating from the aorta
	1	LHA originating from CHA
Yaprak O	3	RHA originating from CA
	2	Trifurcation^a^
	2	Accessory RHA originating from GDA
	1	RHA originating from the aorta and accessory LHA originating from LGA
	1	CA bifurcating into different branches accompanied by replaced RHA originating from SMA
Skorzewska A	1	RHA originating from the aorta and LHA originating from LGA
	1	Double hepatic artery^b^
	1	LHA originating from GDA
Araujo Neto SA	2	RHA originating from CA
	2	LHA originating from CHA
	2	Trifurcation^a^
Nemeth K	3	Trifurcation^a^
	1	RHA originating from the proximal part of CHA and CHA trifurcationg into LHA, GDA, and RGA
	1	Pentafurcation^c^
	1	RHA originating from CA
	1	RHA originating from the proximal part of CHA
	1	RHA-PB originating from CHA
	1	LHA originating from the proximal part of CHA
Thangarajah A	7	double hepatic artery
	11	Trifurcation^a^

*CA* celiac axis, *GDA* gastroduodenal artery, *LHA* left hepatic artery, *LGA* left gastric artery, *RGA* right gastric artery, *SMA* superior mesenteric artery, *CHA* common hepatic artery

^a^“Trifurcation” means CHA trifurcating into RHA, LHA, and GDA

^b^“Double hepatic artery” means early branching of RHA and LHA from CA

^c^“Pentafurcation” means CHA branching into five arteries, LHA, RHA, artery of segment IV, GDA, and RGA

We performed PD successfully with the resection of this aberrant artery. The optimal surgical strategy in patients with aberrant RHA is under discussion. El Amrani et al. reported that aberrant RHA was preserved in most cases (87%) [1]. Postoperative and oncological outcomes seemed unaffected by RHA variation provided that the aberrant RHA was identified and correctly managed intraoperatively. Okada et al. reported PD was a feasible and safe surgical modality in patients with replaced RHA similar to patients with normal variants [9]. However, it was technically and oncologically difficult to achieve sufficient surgical margins for pancreatic cancer in patients with replaced RHA, regardless of whether there was tumor abutment. They suggested that replaced RHA whose root was situated within 10 mm from tumor should be divided to improve the rate of R0 resection with preoperative therapy (i.e., coil embolization of replaced RHA). Jah et al. reported that aberrant RHA could have three different anatomical courses in relation to the head of the pancreas: posterior route with respect to the head of the pancreas (type I), intraparenchymal route (type II), and deeper route in the superior mesenteric venous groove (type III) [10]. Intraparenchymal aberrant RHA (type II) and those which were involved by tumor should require en bloc resection of the vessel. They also recommended reconstruction in patients where aberrant RHA was large or was thought to represent replaced RHA in en bloc resection of the vessel.

In these chaotic situations, we suggest the following strategy for aberrant RHA in PD. When there is aberrant RHA in preoperative image, you should assess the route with respect to the head of the pancreas and the position of tumor. When aberrant RHA is type I or type III, or the tumor is situated more than 10 mm from aberrant RHA [9], aberrant RHA may be preserved. When aberrant RHA is classified to the other case, en bloc resection of aberrant RHA should be performed. Preoperative coil embolization or reconstruction may be recommended in patient whose aberrant RHA is large or has no collaterals, or liver function is abnormal. In our case, because the tumor was located near the course of RHA-PB at the pancreatic head, RHA-PB was sacrificed. After clamping test, we successfully divided this artery without reconstruction. It might be safe to perform preoperative angiography to know the direction of blood flow of the junction between RHA-PB and PHA-AB because RHA-PB was relatively large. If necessary, we might consider preoperative coil embolization or reconstruction. Careful preoperative assessment of hepatic blood supply and meticulous intraoperative dissection are the keys to perform PD successfully even in this troublesome situation.

## Conclusions

We experienced rare variation of hepatic artery during the resection of the pancreatic head tumor; RHA-PB originating from PIPDA. Careful preoperative assessment of hepatic artery system and its relationship with tumor and the head of pancreas is the key to perform successful PD even in this troublesome case.

## Abbreviations

AB: Anterior branch
PB: Posterior branch
PD: Pancreaticoduodenectomy
PIPDA: Posterior inferior pancreaticoduodenal artery
RHA: Right hepatic artery
